# Supplementary material for: Three-dimensional growth of breast cancer cells potentiates the anti-tumor effects of unacylated ghrelin and AZP-531
Source: eLife. 2020 Jul 15;9:e56913. doi: 10.7554/eLife.56913 (PMC7363447; doi:10.7554/eLife.56913)
Supplement: Figure 2—source data 2. [file elife-56913-fig2-data2.pdf]

Figure 2

b

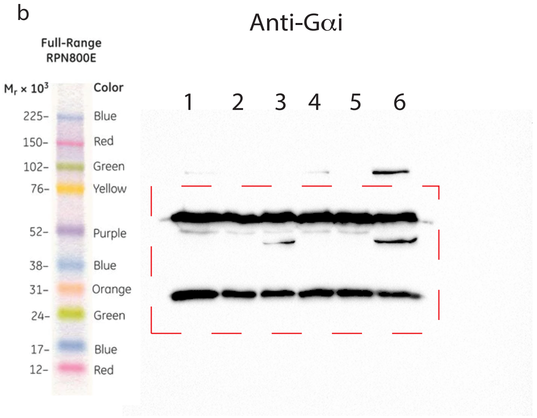

Condition:
Lane 1: Vehicle control 1 (-)_Time 0
Lane 2: UAG_Time 1hr
Lane 3: UAG_Time 2hrs
Lane 4: UAG_Time 6hrs
Lane 5: Melatonin_Time 0.5hr
Lane 6: Melatonin_Time 2hr

MW of Gαi 40 kDa
The indicated cropped area of the blot is shown in the manuscript data.

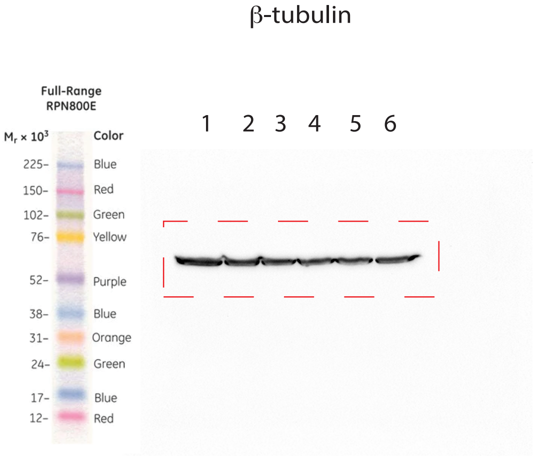

Condition:
Lane 1: Vehicle control 1 (-)_Time 0
Lane 2: UAG_Time 1hr
Lane 3: UAG_Time 2hrs
Lane 4: UAG_Time 6hrs
Lane 5: Melatonin_Time 0.5hr
Lane 6: Melatonin_Time 2hr

MW of β-tubulin 55 kDa
The indicated cropped area of the blot is shown in the manuscript data.

The precipitated active Gαi was immunoblotted with an anti-Gαi antibody . Bound antibodies were revealed with HRP conjugated secondary antibodies (1:2000) using SuperSignal West pico chemiluminescent solution (Pierce, Rockford, IL). The same blot was then stripped and reprobed with β–tubulin ; used as a control).
