# Supplementary material for: Three-dimensional growth of breast cancer cells potentiates the anti-tumor effects of unacylated ghrelin and AZP-531
Source: eLife. 2020 Jul 15;9:e56913. doi: 10.7554/eLife.56913 (PMC7363447; doi:10.7554/eLife.56913)
Supplement: Figure 3—source data 2. [file elife-56913-fig3-data2.pdf]

Figure 3

b

Rabbit Polyclonal anti-phospho-p44/42 MAPK (Erk1/2) (Thr202/Tyr204)

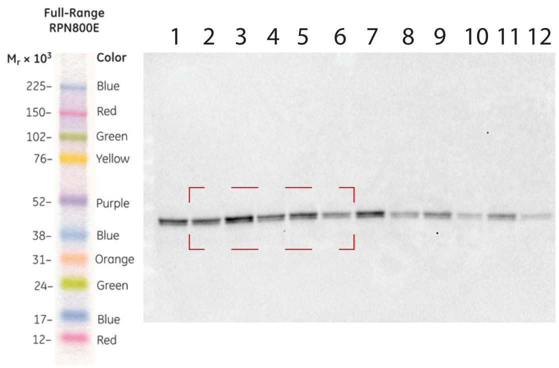

Condition:
Lane 1: Vehicle control 1 (-)_Time 0
Lane 2: Vehicle control 2 (-)_Time 0
Lane 3: 10% FBS (+)_Time 5 mins
Lane 4: UAG+ 10% FBS_Time 5 mins
Lane 5: 10% FBS (+)_Time 15 mins
Lane 6: UAG+ 10% FBS_Time 15 mins
Lane 7: 10% FBS (+)_Time 30 mins
Lane 8: UAG+ 10% FBS_Time 30 mins
Lane 9: 10% FBS (+)_Time 60 mins
Lane 10: UAG+ 10% FBS_Time 60 mins
Lane 11: 10% FBS (+)_Time 120 mins
Lane 12: UAG+ 10% FBS_Time 120 mins

MW of pERK: 42,44 kDa
The indicated cropped area of the blot is shown in the manuscript data.

Rabbit Monoclonal anti-p44/42 MAPK (Erk1/2) (137F5)

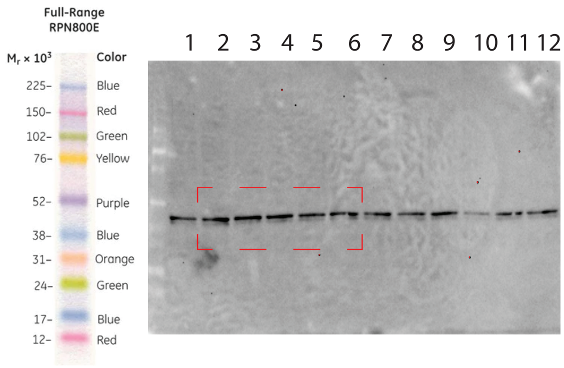

Condition:
Lane 1: Vehicle control 1 (-)_Time 0
Lane 2: Vehicle control 2 (-)_Time 0
Lane 3: 10% FBS (+)_Time 5 mins
Lane 4: UAG+ 10% FBS_Time 5 mins
Lane 5: 10% FBS (+)_Time 15 mins
Lane 6: UAG+ 10% FBS_Time 15 mins
Lane 7: 10% FBS (+)_Time 30 mins
Lane 8: UAG+ 10% FBS_Time 30 mins
Lane 9: 10% FBS (+)_Time 60 mins
Lane 10: UAG+ 10% FBS_Time 60 mins
Lane 11: 10% FBS (+)_Time 120 mins
Lane 12: UAG+ 10% FBS_Time 120 mins

MW of tERK: 42,44 kDa
The indicated cropped area of the blot is shown in the manuscript data.

We first probed the blot with Rabbit Polyclonal anti-phospho-p44/42 MAPK (Erk1/2) (Thr202/Tyr204) (detected endogenous levels of p44 and p42 MAP Kinase (Erk1 and Erk2) when phosphorylated either individually or dually at Thr202 and Tyr204 of Erk1 (Thr185 and Tyr187 of Erk2). The same blot was then stripped and reprobed with Rabbit Monoclonal anti-p44/42 MAPK (Erk1/2) (137F5) (detected endogenous levels of total p44/42 MAP kinase (Erk1/Erk2) protein; used as a control).

Figure 3

b

Rabbit Monoclonal anti-phospho-p90RSK (Ser380) (D3H11)

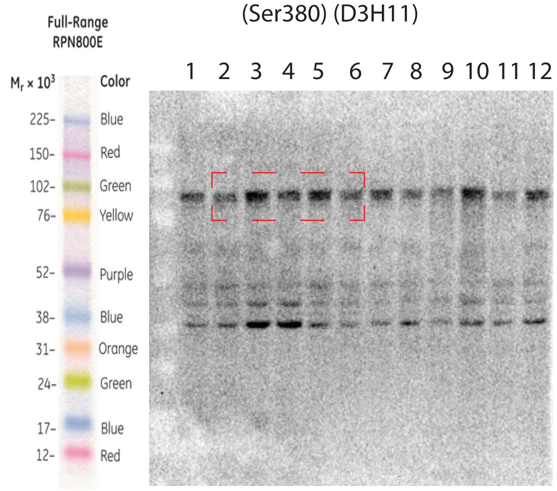

Condition:
Lane 1: Vehicle control 1 (-)_Time 0
Lane 2: Vehicle control 2 (-)_Time 0
Lane 3: 10% FBS (+)_Time 5 mins
Lane 4: UAG+ 10% FBS_Time 5 mins
Lane 5: 10% FBS (+)_Time 15 mins
Lane 6: UAG+ 10% FBS_Time 15 mins
Lane 7: 10% FBS (+)_Time 30 mins
Lane 8: UAG+ 10% FBS_Time 30 mins
Lane 9: 10% FBS (+)_Time 60 mins
Lane 10: UAG+ 10% FBS_Time 60 mins
Lane 11: 10% FBS (+)_Time 120 mins
Lane 12: UAG+ 10% FBS_Time 120 mins

MW of p-p90RSK: 90 kDa
The indicated cropped area of the blot is shown in the manuscript data.

Rabbit Monoclonal anti-RSK1/RSK2/RSK3 (32D7)

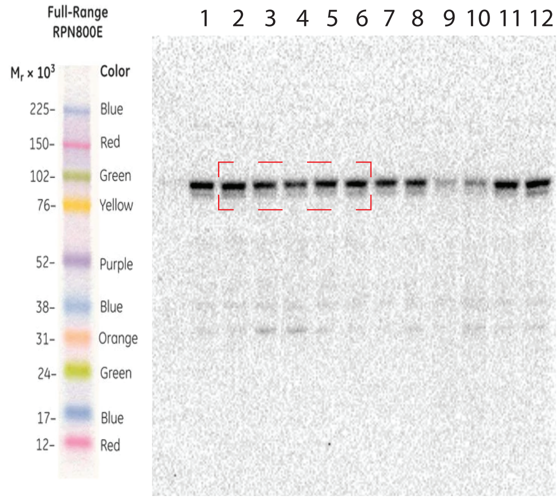

Condition:
Lane 1: Vehicle control 1 (-)_Time 0
Lane 2: Vehicle control 2 (-)_Time 0
Lane 3: 10% FBS (+)_Time 5 mins
Lane 4: UAG+ 10% FBS_Time 5 mins
Lane 5: 10% FBS (+)_Time 15 mins
Lane 6: UAG+ 10% FBS_Time 15 mins
Lane 7: 10% FBS (+)_Time 30 mins
Lane 8: UAG+ 10% FBS_Time 30 mins
Lane 9: 10% FBS (+)_Time 60 mins
Lane 10: UAG+ 10% FBS_Time 60 mins
Lane 11: 10% FBS (+)_Time 120 mins
Lane 12: UAG+ 10% FBS_Time 120 mins

MW of tRSK: 90 kDa
The indicated cropped area of the blot is shown in the manuscript data.

We first probed the blot with Rabbit Monoclonal anti-phospho-p90RSK (Ser380) (D3H11) (recognized endogenous levels of p90RSK1 protein when phosphorylated at Ser380. This antibody also detected p90RSK2 phosphorylated at Ser386 and p90RSK3 phosphorylated at Ser377) . The same blot was then stripped and reprobed with Rabbit Monoclonal anti-RSK1/RSK2/RSK3 (32D7) (detected endogenous levels of total RSK1/RSK2/RSK3 proteins; used as a control).

c

Rabbit Monoclonal anti-c-Myc (D84C12)

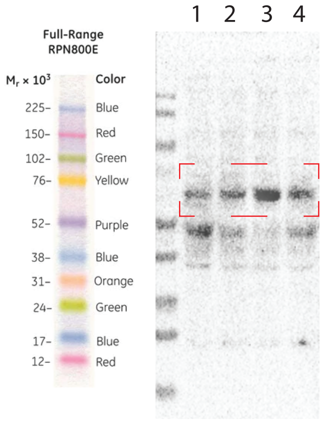

Condition:
Lane 1: Vehicle control (-)
Lane 2: UAG
Lane 3: 10% FBS (+)
Lane 4: UAG+ 10% FBS

MW of c-MYC: 57-65kDa

Mouse Monoclonal anti-β-Actin–Peroxidase

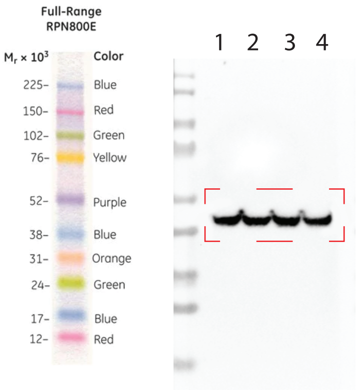

Condition:
Lane 1: Vehicle control (-)
Lane 2: UAG
Lane 3: 10% FBS (+)
Lane 4: UAG+ 10% FBS

MW of β-Actin: 42kDa

We first probed the blot with Rabbit Monoclonal anti-c-Myc (D84C12) (detected endogenous levels of total c-Myc protein).
The same blot was then stripped and reprobed with Mouse Monoclonal anti-β-Actin–Peroxidase (used as a loading control).
The indicated cropped area of the blot is shown in the manuscript data.

Figure 3

d

Rabbit Polyclonal anti-phospho-Akt (Ser473)

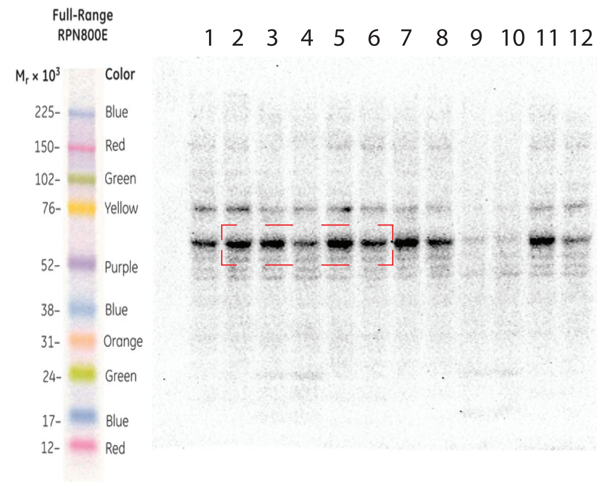

Condition:
Lane 1: Vehicle control 1 (-)_Time 0
Lane 2: Vehicle control 2 (-)_Time 0
Lane 3: 10% FBS (+)_Time 5 mins
Lane 4: UAG+ 10% FBS_Time 5 mins
Lane 5: 10% FBS (+)_Time 15 mins
Lane 6: UAG+ 10% FBS_Time 15 mins
Lane 7: 10% FBS (+)_Time 30 mins
Lane 8: UAG+ 10% FBS_Time 30 mins
Lane 9: 10% FBS (+)_Time 60 mins
Lane 10: UAG+ 10% FBS_Time 60 mins
Lane 11: 10% FBS (+)_Time 120 mins
Lane 12: UAG+ 10% FBS_Time 120 mins

MW of pAKT: 60 kDa
The indicated cropped area of the blot is shown in the manuscript data.

Rabbit Polyclonal anti-Akt

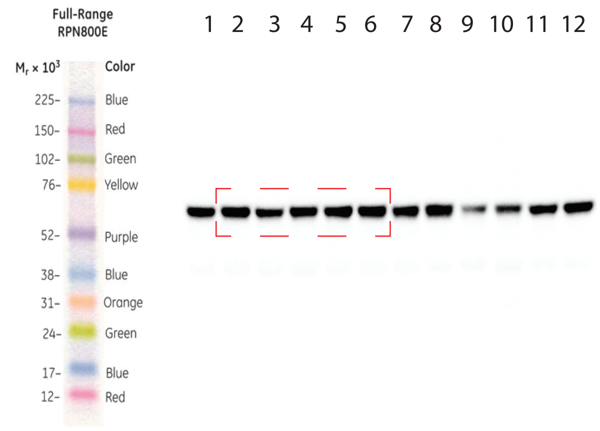

Condition:
Lane 1: Vehicle control 1 (-)_Time 0
Lane 2: Vehicle control 2 (-)_Time 0
Lane 3: 10% FBS (+)_Time 5 mins
Lane 4: UAG+ 10% FBS_Time 5 mins
Lane 5: 10% FBS (+)_Time 15 mins
Lane 6: UAG+ 10% FBS_Time 15 mins
Lane 7: 10% FBS (+)_Time 30 mins
Lane 8: UAG+ 10% FBS_Time 30 mins
Lane 9: 10% FBS (+)_Time 60 mins
Lane 10: UAG+ 10% FBS_Time 60 mins
Lane 11: 10% FBS (+)_Time 120 mins
Lane 12: UAG+ 10% FBS_Time 120 mins

MW of t-AKT: 60 kDa
The indicated cropped area of the blot is shown in the manuscript data.

We first probed the blot with Rabbit Polyclonal anti-phospho-Akt (Ser473) (detected endogenous level of AKT only when phosphorylated Ser473). The same blot was then stripped and reprobed with Rabbit Polyclonal anti-Akt (detected endogenous level of AKT1, AKT2, AKT3 proteins; used as a control).

Figure 3

d

Rabbit Monoclonal anti-phospho-p70 S6 Kinase (Thr389) (108D2)

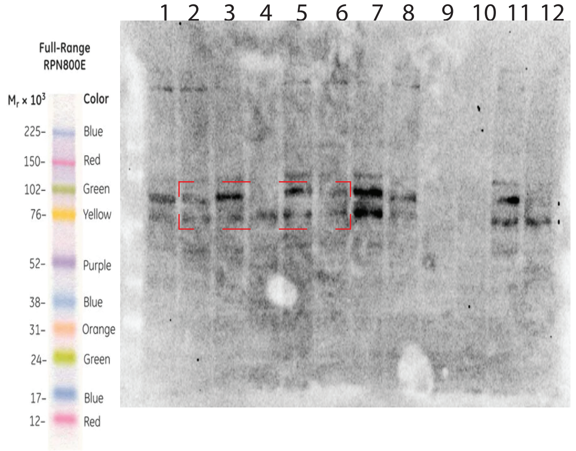

Condition:
Lane 1: Vehicle control 1 (-)_Time 0
Lane 2: Vehicle control 2 (-)_Time 0
Lane 3: 10% FBS (+)_Time 5 mins
Lane 4: UAG+ 10% FBS_Time 5 mins
Lane 5: 10% FBS (+)_Time 15 mins
Lane 6: UAG+ 10% FBS_Time 15 mins
Lane 7: 10% FBS (+)_Time 30 mins
Lane 8: UAG+ 10% FBS_Time 30 mins
Lane 9: 10% FBS (+)_Time 60 mins
Lane 10: UAG+ 10% FBS_Time 60 mins
Lane 11: 10% FBS (+)_Time 120 mins
Lane 12: UAG+ 10% FBS_Time 120 mins

MW of p-p70S6K: 70,85 kDa
The indicated cropped area of the blot is shown in the manuscript data.

Rabbit Monoclonal anti-p70 S6 Kinase (49D7)

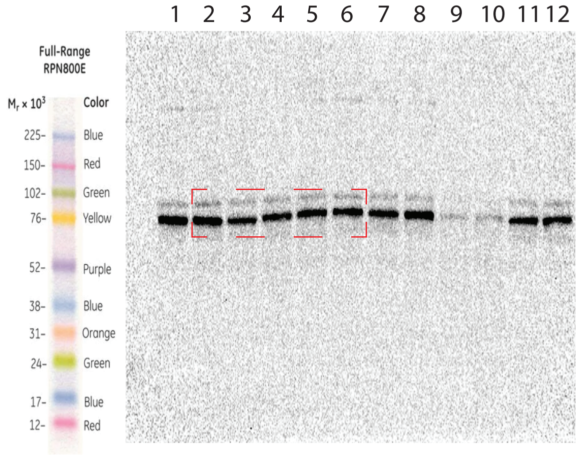

Condition:
Lane 1: Vehicle control 1 (-)_Time 0
Lane 2: Vehicle control 2 (-)_Time 0
Lane 3: 10% FBS (+)_Time 5 mins
Lane 4: UAG+ 10% FBS_Time 5 mins
Lane 5: 10% FBS (+)_Time 15 mins
Lane 6: UAG+ 10% FBS_Time 15 mins
Lane 7: 10% FBS (+)_Time 30 mins
Lane 8: UAG+ 10% FBS_Time 30 mins
Lane 9: 10% FBS (+)_Time 60 mins
Lane 10: UAG+ 10% FBS_Time 60 mins
Lane 11: 10% FBS (+)_Time 120 mins
Lane 12: UAG+ 10% FBS_Time 120 mins

MW of t-p70S6K: 70,85 kDa
The indicated cropped area of the blot is shown in the manuscript data.

We first probed the blot with Rabbit Monoclonal anti-phospho-p70 S6 Kinase (Thr389) (108D2) (detected endogenous levels of p70 S6 kinase only when phosphorylated at Thr389. This antibody also detected p85 S6 kinase when phosphorylated at the analogous site (Thr412) and possibly S6KII phosphorylated at Thr401). The same blot was then stripped and reprobed with Rabbit Monoclonal anti-p70 S6 Kinase (49D7) (detected endogenous levels of total p70 S6 kinase protein. The antibody also recognizedp85 S6 kinase; used as a control).
