# Supplementary material for: Three-dimensional growth of breast cancer cells potentiates the anti-tumor effects of unacylated ghrelin and AZP-531
Source: eLife. 2020 Jul 15;9:e56913. doi: 10.7554/eLife.56913 (PMC7363447; doi:10.7554/eLife.56913)
Supplement: Figure 4—source data 2. [file elife-56913-fig4-data2.pdf]

Figure 4

g

Rabbit Monoclonal anti-CDK4 (D9G3E)

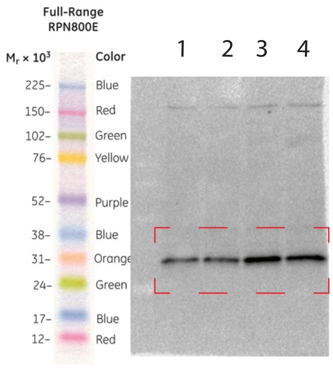

Condition:
Lane 1: Vehicle control (-)
Lane 2: UAG
Lane 3: 10% FBS (+)
Lane 4: UAG+ 10% FBS

MW of CDK4: 30kDa

Mouse Monoclonal anti-β-Actin–Peroxidase

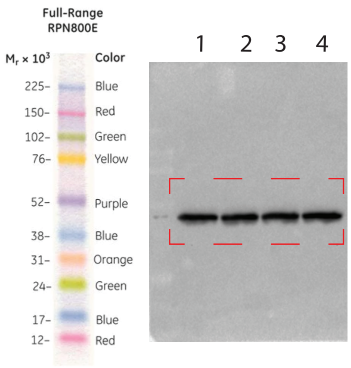

Condition:
Lane 1: Vehicle control (-)
Lane 2: UAG
Lane 3: 10% FBS (+)
Lane 4: UAG+ 10% FBS

MW of β-Actin: 42kDa

We first probed the blot with Rabbit Monoclonal anti-CDK4 (D9G3E) (detected endogenous levels of total CDK4 protein). The same blot was then stripped and reprobed with Mouse Monoclonal anti-β-Actin–Peroxidase (used as a loading control). The indicated cropped area of the blot is shown in the manuscript data.

Mouse Monoclonal anti-Cyclin D3 (DCS22)

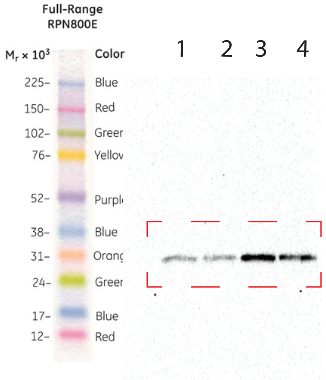

Condition:
Lane 1: Vehicle control (-)
Lane 2: UAG
Lane 3: 10% FBS (+)
Lane 4: UAG+ 10% FBS

MW of Cyclin D3: 31kDa

Mouse Monoclonal anti-β-Actin–Peroxidase

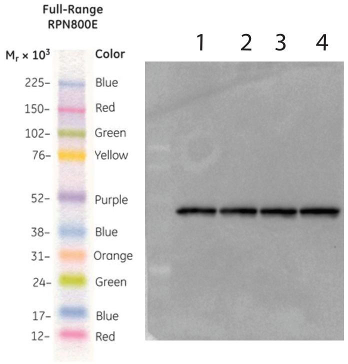

Condition:
Lane 1: Vehicle control (-)
Lane 2: UAG
Lane 3: 10% FBS (+)
Lane 4: UAG+ 10% FBS

MW of β-Actin: 42kDa

We first probed the blot with Mouse Monoclonal anti-Cyclin D3 (DCS22) (detected endogenous levels of total Cyclin D3 protein). The same blot was then stripped and reprobed with Mouse Monoclonal anti-β-Actin–Peroxidase (used as a loading control). The indicated cropped area of the blot is shown in the manuscript data.

Rabbit Polyclonal anti-phospho-Rb (Ser795)

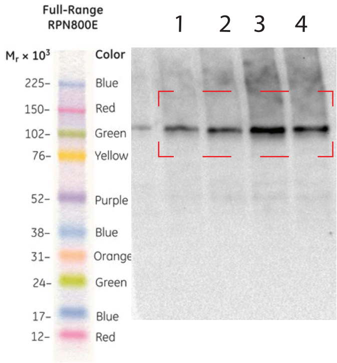

Condition:
Lane 1: Vehicle control (-)
Lane 2: UAG
Lane 3: 10% FBS (+)
Lane 4: UAG+ 10% FBS

MW of pRB (Ser795) : 110kDa

Mouse Monoclonal anti-Rb (4H1)

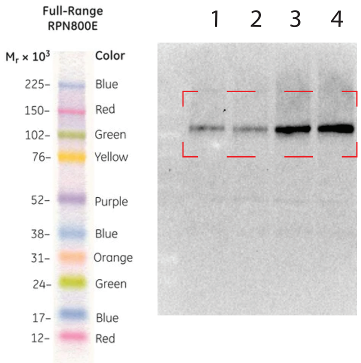

Condition:
Lane 1: Vehicle control (-)
Lane 2: UAG
Lane 3: 10% FBS (+)
Lane 4: UAG+ 10% FBS

MW of RB: 110kDa

We first probed the blot with Rabbit Polyclonal anti-phospho-Rb (Ser795) (detected endogenous levels of Rb only when phosphorylated at Ser795).The same blot was then stripped and reprobed with Mouse Monoclonal anti-Rb (4H1) (detected endogenous levels of total Rb protein;used as a control). The indicated cropped area of the blot is shown in the manuscript data.

Figure 4

g

Rabbit Monoclonal anti-Bcl-2 (D55G8)
(Human Specific)

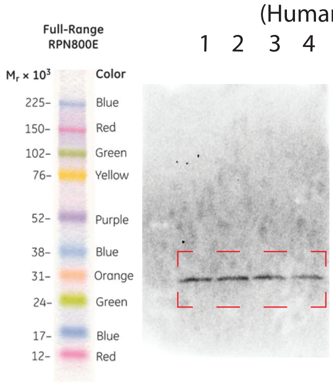

Condition:
Lane 1: Vehicle control (-)
Lane 2: UAG
Lane 3: 10% FBS (+)
Lane 4: UAG+ 10% FBS

MW of Bcl2: 28kDa

Mouse Monoclonal anti-β-Actin–Peroxidase

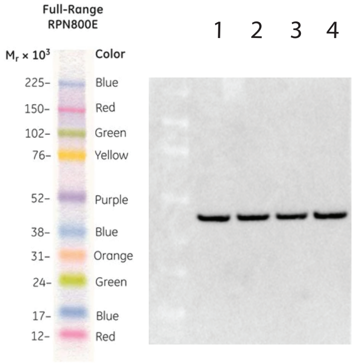

Condition:
Lane 1: Vehicle control (-)
Lane 2: UAG
Lane 3: 10% FBS (+)
Lane 4: UAG+ 10% FBS

MW of β-Actin: 42kDa

We first probed the blot with Rabbit Monoclonal anti-Bcl-2 (D55G8) (Human Specific) (detected endogenous levels of Bcl-2 only when phosphorylated at threonine 56. The same blot was then stripped and reprobed with Mouse Monoclonal anti-β-Actin–Peroxidase (used as a loading control). The indicated cropped area of the blot is shown in the manuscript data.

Rabbit Monoclonal anti-Bax

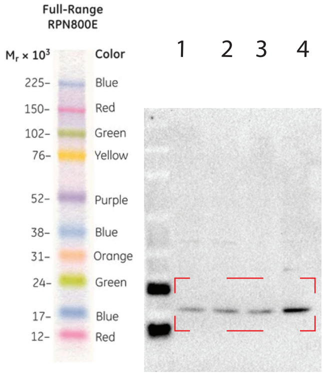

Condition:
Lane 1: Vehicle control (-)
Lane 2: UAG
Lane 3: 10% FBS (+)
Lane 4: UAG+ 10% FBS

MW of Bax: 20kDa

Mouse Monoclonal anti-β-Actin–Peroxidase

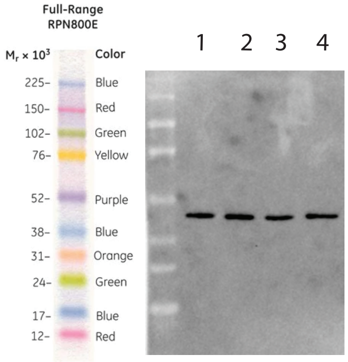

Condition:
Lane 1: Vehicle control (-)
Lane 2: UAG
Lane 3: 10% FBS (+)
Lane 4: UAG+ 10% FBS

MW of β-Actin: 42kDa

We first probed the blot with Rabbit Monoclonal anti-Bax (detected endogenous levels of total Bax protein).
The same blot was then stripped and reprobed with Mouse Monoclonal anti-β-Actin–Peroxidase (used as a loading control).
The indicated cropped area of the blot is shown in the manuscript data.

Figure 4

h

Rabbit Monoclonal anti-CDK4 (D9G3E)

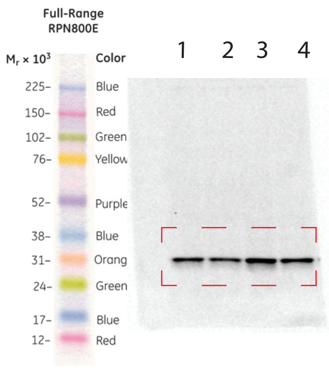

Condition:
Lane 1: Vehicle control (-)
Lane 2: UAG
Lane 3: 10% FBS (+)
Lane 4: UAG+ 10% FBS

MW of CDK4: 30kDa

Mouse Monoclonal anti-β-Actin–Peroxidase

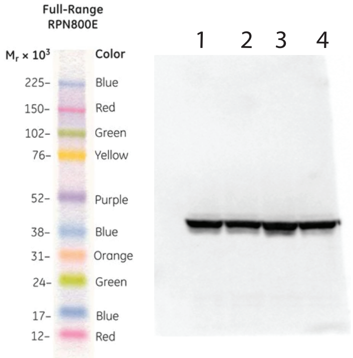

Condition:
Lane 1: Vehicle control (-)
Lane 2: UAG
Lane 3: 10% FBS (+)
Lane 4: UAG+ 10% FBS

MW of β-Actin: 42kDa

We first probed the blot with Rabbit Monoclonal anti-CDK4 (D9G3E) (detected endogenous levels of total CDK4 protein). The same blot was then stripped and reprobed with Mouse Monoclonal anti-β-Actin–Peroxidase (used as a loading control). The indicated cropped area of the blot is shown in the manuscript data.

Mouse Monoclonal anti-Cyclin D3 (DCS22)

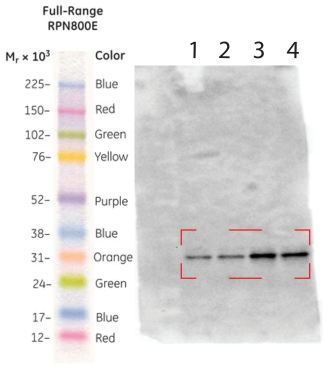

Condition:
Lane 1: Vehicle control (-)
Lane 2: UAG
Lane 3: 10% FBS (+)
Lane 4: UAG+ 10% FBS

MW of Cyclin D3: 31kDa

Mouse Monoclonal anti-β-Actin–Peroxidase

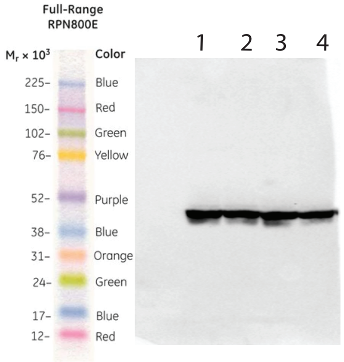

Condition:
Lane 1: Vehicle control (-)
Lane 2: UAG
Lane 3: 10% FBS (+)
Lane 4: UAG+ 10% FBS

MW of β-Actin: 42kDa

We first probed the blot with Mouse Monoclonal anti-Cyclin D3 (DCS22) (detected endogenous levels of total Cyclin D3 protein). The same blot was then stripped and reprobed with Mouse Monoclonal anti-β-Actin–Peroxidase (used as a loading control). The indicated cropped area of the blot is shown in the manuscript data.

Rabbit Polyclonal anti-phospho-Rb (Ser795)

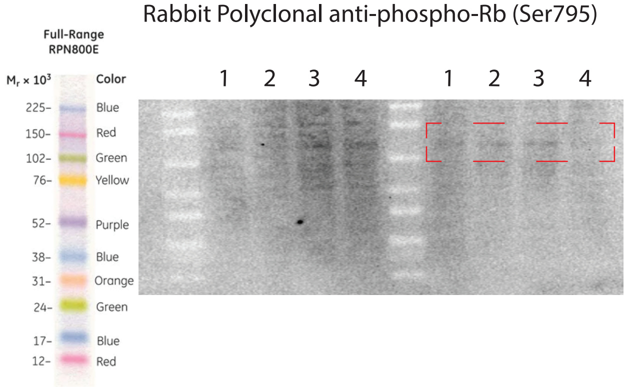

Condition:
Lane 1: Vehicle control (-)
Lane 2: UAG
Lane 3: 10% FBS (+)
Lane 4: UAG+ 10% FBS

MW of pRB (Ser795) : 110kDa
The indicated cropped area of the blot is shown in the manuscript data.

Mouse Monoclonal anti-Rb (4H1)

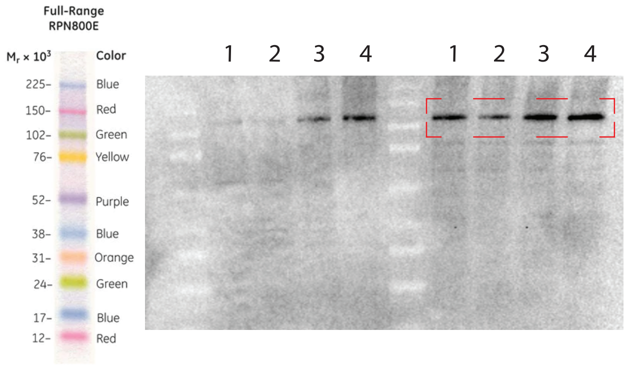

Condition:
Lane 1: Vehicle control (-)
Lane 2: UAG
Lane 3: 10% FBS (+)
Lane 4: UAG+ 10% FBS

MW of RB: 110kDa
The indicated cropped area of the blot is shown in the manuscript data.

We first probed the blot with Rabbit Polyclonal anti-phospho-Rb (Ser795) (detected endogenous levels of Rb only when phosphorylated at Ser795).The same blot was then stripped and reprobed with Mouse Monoclonal anti-Rb (4H1) (detected endogenous levels of total Rb protein;used as a control). The indicated cropped area of the blot is shown in the manuscript data.

Figure 4

h

Rabbit Monoclonal anti-Bcl-2 (D55G8)
(Human Specific)

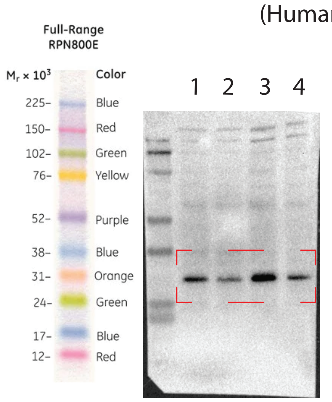

Condition:
Lane 1: Vehicle control (-)
Lane 2: UAG
Lane 3: 10% FBS (+)
Lane 4: UAG+ 10% FBS

MW of Bcl2: 28kDa

Mouse Monoclonal anti-β-Actin–Peroxidase

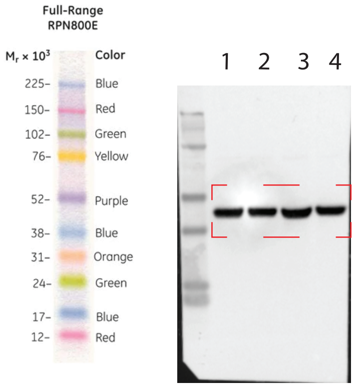

Condition:
Lane 1: Vehicle control (-)
Lane 2: UAG
Lane 3: 10% FBS (+)
Lane 4: UAG+ 10% FBS

MW of β-Actin: 42kDa

We first probed the blot with Rabbit Monoclonal anti-Bcl-2 (D55G8) (Human Specific) (detected endogenous levels of Bcl-2 only when phosphorylated at threonine 56. The same blot was then stripped and reprobed with Mouse Monoclonal anti-β-Actin–Peroxidase (used as a loading control). The indicated cropped area of the blot is shown in the manuscript data.

Rabbit Monoclonal anti-Bax

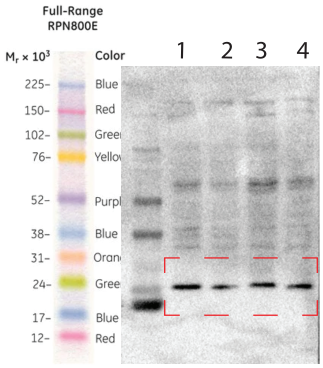

Condition:
Lane 1: Vehicle control (-)
Lane 2: UAG
Lane 3: 10% FBS (+)
Lane 4: UAG+ 10% FBS

MW of Bax: 20kDa

Mouse Monoclonal anti-β-Actin–Peroxidase

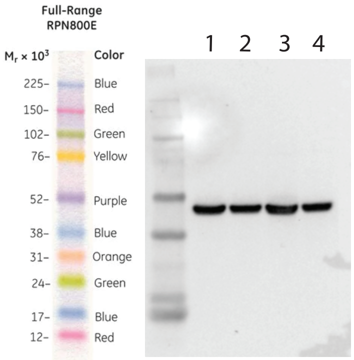

Condition:
Lane 1: Vehicle control (-)
Lane 2: UAG
Lane 3: 10% FBS (+)
Lane 4: UAG+ 10% FBS

MW of β-Actin: 42kDa

We first probed the blot with Rabbit Monoclonal anti-Bax (detected endogenous levels of total Bax protein).
The same blot was then stripped and reprobed with Mouse Monoclonal anti-β-Actin–Peroxidase (used as a loading control).
The indicated cropped area of the blot is shown in the manuscript data.
