# Supplementary material for: Three-dimensional growth of breast cancer cells potentiates the anti-tumor effects of unacylated ghrelin and AZP-531
Source: eLife. 2020 Jul 15;9:e56913. doi: 10.7554/eLife.56913 (PMC7363447; doi:10.7554/eLife.56913)
Supplement: Supplementary file 1. [file elife-56913-supp1.docx]

| sequence-based reagent | GNAI1A sequence guide strands (Forward) | This paper | PCR primers | CACCGAGCACTGAGTGACTACGACC |
| --- | --- | --- | --- | --- |
| sequence-based reagent | GNAI1A sequence guide strands (Reverse) | This paper | PCR primers | AAACGGTCGTAGTCACTCAGTGCTC |
| sequence-based reagent | GNAI1B sequence guide strands (Forward) | This paper | PCR primers | CACCGTTGCTATCATTAGGGCTATG |
| sequence-based reagent | GNAI1B sequence guide strands (Reverse) | This paper | PCR primers | AAACATAGCCCTAATGATAGCAAC |
| sequence-based reagent | GNAI1C sequence guide strands (Forward) | This paper | PCR primers | CACCGTGAAGCTGGTTATTCAGAAG |
| sequence-based reagent | GNAI1C sequence guide strands (Reverse) | This paper | PCR primers | AAACCTTCTGAATAACCAGCTTCAC |
| sequence-based reagent | GNAI2A sequence guide strands (Forward) | This paper | PCR primers | CACCGGACCCGCGTAAAGACCACG |
| sequence-based reagent | GNAI2A sequence guide strands (Reverse) | This paper | PCR primers | AAACCGTGGTCTTTACGCGGGTCCC |
| sequence-based reagent | GNAI2B sequence guide strands (Forward) | This paper | PCR primers | CACCGCTTTGCCGACCCCTCCAGAG |
| sequence-based reagent | GNAI2B sequence guide strands (Reverse) | This paper | PCR primers | AAACCTCTGGAGGGGTCGGCAAAGC |
| sequence-based reagent | GNAI2C sequence guide strands (Forward) | This paper | PCR primers | CACCGGCGTCATCCGGAGGCTCTGG |
| sequence-based reagent | GNAI2C sequence guide strands (Reverse) | This paper | PCR primers | AAACCCAGAGCCTCCGGATGACGCC |
| sequence-based reagent | GNAI3A sequence guide strands (Forward) | This paper | PCR primers | CACCGGATCGACCGCAACTTACGGG |
| sequence-based reagent | GNAI3A sequence guide strands (Reverse) | This paper | PCR primers | AAACCCCGTAAGTAAGTTGCGGTCGATCC |
| sequence-based reagent | GNAI3B sequence guide strands (Forward) | This paper | PCR primers | CACCGTCATGAGGATGGCTATTCAG |
| sequence-based reagent | GNAI3B sequence guide strands (Reverse) | This paper | PCR primers | AAACCTGAATAGCCATCCTCATGAC |
| sequence-based reagent | GNAI3C sequence guide strands (Forward) | This paper | PCR primers | CACCGAGTCTAACTACATTCCAACT |
| sequence-based reagent | GNAI3C sequence guide strands (Reverse) | This paper | PCR primers | AAACAGTTGGAATGTAGTTAGACTC |
